# Impact of Thyroid Hormone Imbalance on Electrocardiographic Parameters: Systematic Review and Meta-Analysis

**DOI:** 10.3390/jcm14248755

**Published:** 2025-12-10

**Authors:** Maksymilian Kłosowicz, Magdalena Urbańczuk, Aleksandra Burbelka, Agnieszka Gala-Błądzińska, Krzysztof Balawender

**Affiliations:** 1 Department of Normal and Clinical Anatomy, Faculty of Medicine, Medical College, University of Rzeszów, 35-615 Rzeszów, Polandbalawender82@gmail.com (K.B.); 2 Department of Internal Medicine, Nephrology and Endocrinology, St. Queen Jadwiga Clinical Hospital Nr 2 in Rzeszow, 35-301 Rzeszow, Poland; magdalena.amarowicz@wp.pl (M.U.); aggala@ur.edu.pl (A.G.-B.); 3 Department of Nephrology and Endocrinology, Faculty of Medicine, Medical College, University of Rzeszów, 35-310 Rzeszów, Poland

**Keywords:** electrocardiography, thyroid disorders, thyroid hormone imbalance, QT interval, heart rate variability

## Abstract

**Background:** Thyroid dysfunction is a prevalent endocrine disorder with significant cardiovascular consequences, particularly through its effects on cardiac electrophysiology. Electrocardiography (ECG), as a widely available and cost-effective diagnostic tool, provides valuable insight into these alterations. This systematic review and meta-analysis aimed to evaluate the relationship between thyroid hormone imbalance and ECG parameters. **Methods:** A comprehensive search of PubMed, ScienceDirect, and Google Scholar identified 1099 studies, of which 121 underwent full-text analysis. Ultimately, 37 studies with complete datasets were included in the quantitative synthesis, encompassing 167,074 participants across overt hyperthyroidism, subclinical and overt hypothyroidism, and euthyroid control groups. **Results:** Meta-analysis revealed significant alterations in key electrophysiological markers. Overt hyperthyroidism was associated with QTc and Tp-e prolongation, consistent with increased repolarization heterogeneity and arrhythmic risk. In overt hypothyroidism, QTc and Tp-e intervals were also prolonged, accompanied by reduced heart rate variability, reflecting autonomic imbalance. Subclinical forms demonstrated more variable results, though trends toward conduction and repolarization disturbances were observed. Importantly, several studies indicated that levothyroxine therapy or surgical treatment normalized abnormal ECG findings, underscoring their reversible nature. **Conclusions:** These results highlight the strong association between thyroid hormone abnormalities and ECG alterations, which may serve as early markers of arrhythmic risk and sudden cardiac death. Incorporating ECG screening into thyroid disease management could improve early detection, risk stratification, and cardiovascular prevention strategies.

## 1. Introduction

Population-based research involving more than 50,000 participants has demonstrated that thyroid dysfunction occurs in about 5% of adults, disproportionately affecting women and the elderly [1]. In another study by the same team, involving 33,000 patients between 2007 and 2012, the prevalence of subclinical hypothyroidism (SCH) was 4.3%, overt hypothyroidism (CH) 0.33%, subclinical hyperthyroidism (SCHyper) 3.2%, and overt hyperthyroidism (CHyper) 0.2% [2]. The prevalence of these disorders is also influenced by ethnicity—in a study conducted in Mexico, SCH was present in 5% of participants, CH in 1.8%, and SCHyper in 2.8% [3].

Thyroid hormones (THs) play a crucial role in regulating various body systems, one of the most sensitive being the cardiovascular system (CVS). Thyroid hormone receptors (TRs) are present both in the myocardium and in the walls of blood vessels [4]. Their presence within cardiovascular structures means that changes in thyroid hormone levels can lead to numerous complications—including heart failure, both systolic and diastolic hypertension, arrhythmia, myocardial structural abnormalities, coronary artery disease, and thromboembolic events. THs also influence lipid metabolism, contributing to the development of atherosclerosis and thrombogenesis [5,6,7,8]. Two receptor isoforms dominate in the CVS: TRα1, found mainly in working ventricular cardiomyocytes and peripheral conduction pathways, and TRβ1, present primarily in conduction system cells. Both isoforms are co-expressed in the atria and proximal conduction structures (SA and AV nodes) [9,10]. THs additionally regulate the expression of β1-adrenergic and muscarinic receptors, further modulating cardiac function.

Thyroid hormones act through both genomic and non-genomic pathways [11]. Genomic effects occur via nuclear receptors and modify gene expression, influencing cardiomyocyte function, metabolism, growth, and ion channel protein expression [12,13,14]. Non-genomic actions involve direct modulation of membrane ion channels, affecting myocardial contractility through altered transport of ions, glucose, and amino acids [14].

Abnormal TH levels have also been shown to affect the cardiac conduction system (CCS), which may manifest as changes in the electrocardiogram (ECG) [4,5]. ECG is a widely accessible, quick, and cost-effective examination that provides valuable prognostic information—including insights into the risk of arrhythmias or ischemic stroke. Despite the established impact of thyroid hormones on the cardiac conduction system, relatively few publications focus on their influence on specific ECG parameters. Given the high prevalence and significant clinical implications of thyroid disorders, this review focuses primarily on hyperthyroidism and hypothyroidism, both in overt and subclinical forms.

This systematic review aims to assess the impact of thyroid dysfunction on specific ECG parameters, such as the T wave, Tp-e interval, Tp-e/QT and Tp-e/QTc ratios, QT and QTc intervals, QRS complex morphology, PQ interval, and RR interval, which defines heart rate (HR). Correlating ECG changes with thyroid hormone (TH) levels may, in the future, allow for more accurate risk assessment of arrhythmia occurrence. Early identification of patients at increased risk could contribute to more effective prevention and thus reduce the number of major cardiovascular events such as ischemic stroke or sudden cardiac death.

## 2. Methodology

### 2.1. Study Protocol

**Scheme 1:**
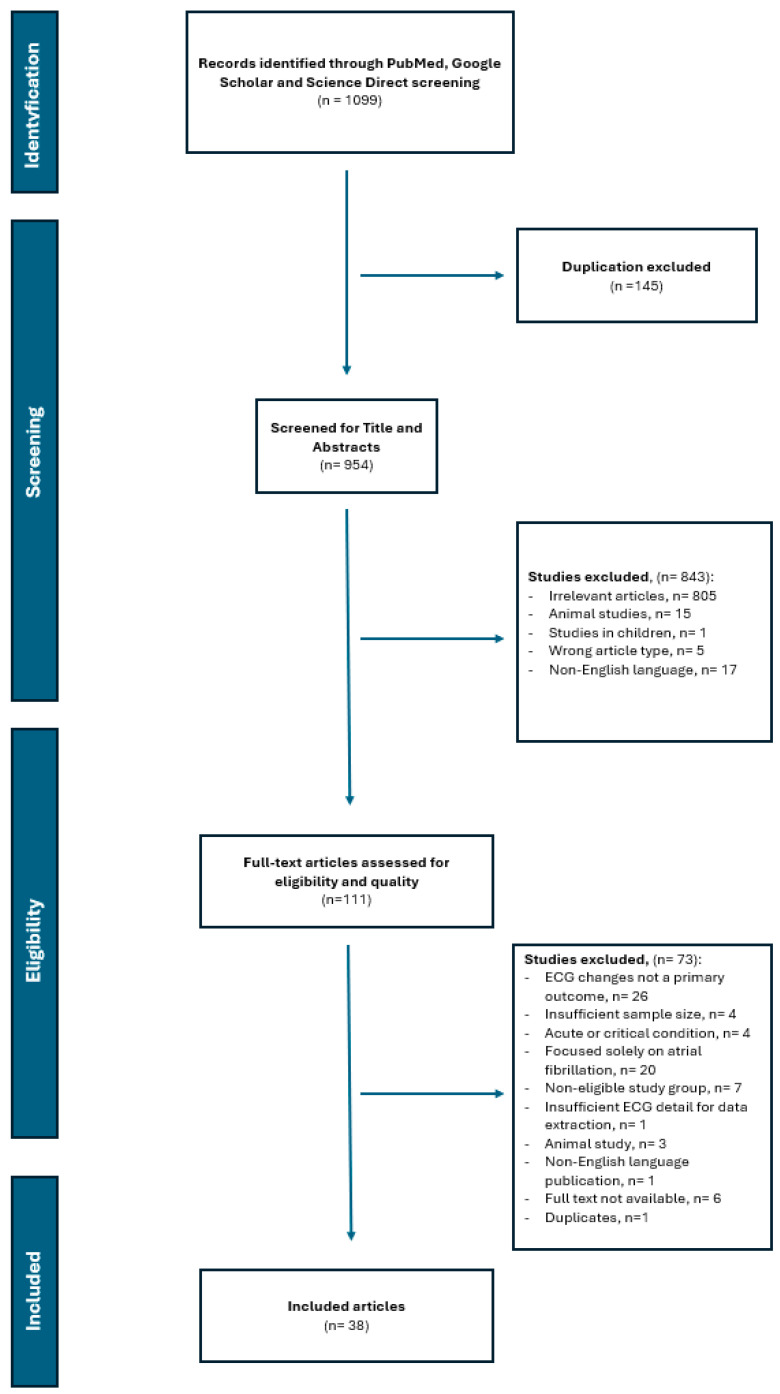
PRISMA Flow Diagram of the Study Selection Process.

The selection process followed the PRISMA (Preferred Reporting Items for Systematic Reviews and Meta-Analyses) 2020 guidelines (Supplementary Table S1) and is illustrated in the PRISMA flow diagram (Scheme 1) [15]. The analysis followed PRISMA recommendations; however, due to the exploratory character of the project and its reliance on routinely reported clinical parameters, a formal review protocol had not been developed prior to data extraction. As no protocol existed, no amendments were applicable.

### 2.2. Literature Search and Search Strategy

The primary bibliographic database used in this review was PubMed, which formed the core of the structured literature search. In addition, ScienceDirect (Elsevier) and Google Scholar were used as supplementary search platforms to identify additional potentially relevant studies and to retrieve full-text articles. ScienceDirect and Google Scholar were not treated as formal bibliographic databases due to their broader and less standardized search algorithms; instead, they served to enhance the sensitivity of the search. Due to the varying indexing mechanisms, available Boolean operators, and filtering capabilities of each database, the search strategy was individually tailored to the specific features of each platform. The search was limited to publications published between 2015 and 2025.

In PubMed, an advanced search strategy was applied, combining Medical Subject Headings (MeSH) terms with Title/Abstract (tiab) searches. In ScienceDirect and Google Scholar, keyword-based searches were employed, aligned with the topic of the review and adapted to the structural and logical operator differences specific to each database. Due to limitations in the use of Boolean operators in ScienceDirect, the search process was repeated three times, each focusing on different electrocardiographic parameters, while consistently applying the same thyroid dysfunction-related keywords. A detailed summary of the keywords used, as well as the Boolean and restrictive operators applied in each database, is presented in Table 1. The last searches were run on 3 July 2025 (PubMed), 3 July 2025 (ScienceDirect), and 3 July 2025 (Google Scholar).

Only English-language publications were included in the review. Duplicate articles, unpublished manuscripts, and conference abstracts were excluded.

After removing duplicates, an initial screening was conducted based on the available titles and abstracts of the retrieved studies. Studies conducted on animal models and those involving patients under the age of 18 were excluded. A total of 121 articles were included for full-text analysis.

### 2.3. Eligibility Criteria

Studies were selected for analysis if they evaluated the impact of thyroid dysfunction on changes in electrocardiographic recordings and disturbances in sinus rhythm variability. Detailed inclusion and exclusion criteria based on full-text analysis are presented in Table 2, in accordance with the PICOS framework.

### 2.4. Selection Process

The initial search using keywords and MeSH terms yielded a total of 1099 results from the three databases. After removing duplicates, 953 unique records remained. A preliminary screening of titles and abstracts was conducted independently by two reviewers using Rayyan (Qatar Computing Research Institute) to enable blinded parallel assessment. Inter-reviewer agreement for this stage was quantified using Cohen’s kappa coefficient, which indicated substantial agreement between reviewers (κ = 0,78). Any disagreements were resolved through discussion and consensus before proceeding to full-text evaluation. Subsequently, all eligible articles were assessed in full-text form, again independently by the same reviewers. Any disagreements regarding article eligibility were resolved through discussion and consensus. When consensus could not be reached, a third reviewer adjudicated.

Predefined inclusion and exclusion criteria, described in the subsection “Eligibility Criteria”, were applied at each stage of the screening process. Studies excluded after full-text assessment, along with reasons for exclusion, are detailed in Scheme 1.

### 2.5. Data Extraction

A clearly defined, standardized data extraction procedure was applied. Data from the selected publications were systematically collected using a pre-designed and piloted data extraction form in Microsoft Excel. Data extraction was performed independently by two reviewers to ensure accuracy and reduce the risk of bias. After independent extraction, the two datasets were compared. Any discrepancies in the extracted information were resolved through discussion and consensus. When consensus could not be reached, a third reviewer adjudicated.

From each article, the following information was extracted: author names, full title, year of publication, country in which the study was conducted, sample size and control group (if applicable), population characteristics (including age and percentage of female participants), type of thyroid dysfunction, and details of any intervention performed.

For the purpose of statistical analysis, additional data were extracted on FT3, FT4, and TSH levels, as well as body mass index (BMI). These values were then compared with specific electrocardiographic parameters or heart rate variability (HRV) measures.

### 2.6. Statistical Analysis

All statistical tests were two-sided, with a significance level set at α = 0.05.

#### 2.6.1. Evaluation of Influential Studies, Outlier Studies, and Publication Bias

All studies within groups underwent verification through these outlier analyses, influence analyses, and publication bias asymmetry assessments, confirming the overall stability of the findings despite noted heterogeneity.

To assess the reliability of the meta-analytic results and identify any studies that might deviate significantly or exert excessive impact, we performed diagnostic evaluations for influence. These evaluations included leave-one-out diagnostic measures for each study, comprising: the studentized residual, the DFFITS statistic, Cook’s distance, the covariance ratio, the leave-one-out residual heterogeneity estimate (τ^2^), the leave-one-out heterogeneity test statistic (Q statistic), DFBETAS values and analyzed the diagonal elements of the hat matrix and the percentage weights assigned to effect sizes or outcomes in the model fitting process.

A study was deemed influential if it satisfied at least one condition: an absolute DFFITS value exceeding 3, with p representing the number of model parameters and k the number of studies; the lower tail probability of a χ^2^ distribution with p degrees of freedom, constrained by Cook’s distance, surpassing 50%; a hat value exceeding 3 × (p/k); or an absolute DFBETAS value greater than 1. Further criteria included studentized residuals beyond ±2.0, Cook’s distances above 1.0, and DFFITS values reflecting significant shifts.

To detect potential outliers within the study set, we applied a criterion where a study was flagged as an outlier if its 95% confidence interval did not intersect with the 95% confidence interval of the overall pooled effect. This approach allowed identification of studies that substantially diverged from the meta-analytic consensus. Publication bias was evaluated through visual assessment of funnel plots and statistical analysis using Egger’s regression test to detect asymmetry.

Studies which a not outlier, non-influential free od significant publication bias (*p* > 0.05) were taken into account for polled results within group of patients.

#### 2.6.2. Risk of Bias Assessment

Risk of bias in the included studies was assessed independently by two reviewers using the Newcastle–Ottawa Scale (NOS) for observational studies. Each study was evaluated across the domains of selection, comparability, and outcome assessment. Discrepancies between reviewers were resolved through discussion until consensus was reached. When needed, a third reviewer adjudicated. The final NOS ratings were used to qualitatively inform interpretation of the findings.

#### 2.6.3. Random Effect Model

The meta-analysis employed a random-effects model to synthesize untransformed means of the outcome measure across studies, utilizing the inverse variance method for weighing each study’s contribution to the pooled estimate. This approach accounts for between-study variability by incorporating a random component, assuming that the true effect sizes vary across studies due to differences in populations or methodologies. The restricted maximum-likelihood (REML) estimator or the Sidik-Jonkman (SJ) estimator was applied to estimate the between-study variance (τ^2^), selected based on its robustness in handling heterogeneity in small to moderate sample size. Confidence intervals for τ^2^ and its square root (τ) were derived using the Q-profile method, which provides a robust approach to quantifying uncertainty in heterogeneity estimates. Heterogeneity was assessed using several metrics: τ^2^, representing the variance of true effects across studies; τ, the standard deviation of these effects; I^2^, indicating the percentage of total variability due to heterogeneity rather than sampling error; and H, the ratio of the total heterogeneity to the within-study variance, all reported with corresponding confidence intervals. The test for heterogeneity was conducted using the Q statistic, and a *p*-value derived from a chi-squared distribution to evaluate the significance of between-study variability.

### 2.7. Characteristics of the Statistical Tool

Analyses were conducted using the R Statistical language (version 4.3.3; R Core Team, 2024) on Windows 11 Pro 64 bit (build 26100), using the packages meta (version 7.0.0), Matrix (version 1.6.5), numDeriv (version 2016.8.1.1), dmetar (version 0.1.0), report (version 0.5.8), metafor (version 4.6.0), and dplyr (version 1.1.4).

## 3. Results

The results comprise two main components: a statistical analysis based on the extracted data and a narrative synthesis derived from the systematic review. The meta-analysis incorporated data from 18 unique studies, with some studies contributing multiple groups, resulting in 39 group-specific entries. The studies included in each group were as follows: overt hyperthyroidism [16,17,18,19,20,21,22]; subclinical hypothyroidism [21,23,24,25,26,27,28,29,30]; overt hypothyroidism [19,21,23,31,32,33]; and control [16,18,19,20,21,22,23,24,25,26,27,28,29,30,31,32,33].

Beyond the quantitative analyses, the systematic review component aimed to qualitatively synthesize the evidence, with particular attention to study design, patient populations, and reported outcomes. 38 articles included in the review were eligible for statistical analysis due to the availability of complete quantitative data.

### 3.1. Demographic Profiles of Participants Across the Overall Cohort and Study Groups

The studies collectively encompassed 167,074 participants, distributed across four thyroid function categories: overt hyperthyroidism (7 studies, N = 1428), subclinical hypothyroidism (9 studies, N = 29,739), overt hypothyroidism (6 studies, N = 576), and control (17 studies, N = 135,331).

These demographic profiles reveal a consistent female predominance in thyroid dysfunction groups, particularly in hypothyroidism, aligning with epidemiological patterns where autoimmune factors may play a role. Ages were generally middle-aged across all groups, with subclinical hypothyroidism showing slightly older participants, potentially reflecting age-related thyroid decline. BMI values indicated a trend toward higher body mass in hypothyroid groups, consistent with metabolic slowing, while hyperthyroid and control groups maintained normal ranges. Overall, the pooled proportion of females was 71.5% (95% CI: 66.0, 77.0; I^2^ = 99.9%), the pooled mean age was 50.7 years (95% CI: 48.7, 52.7; I^2^ = 99.9%), and the pooled mean BMI was 26.2 kg/m^2^ (95% CI: 25.4, 27.0; I^2^ = 99.9%).

In the overt hyperthyroidism group, the pooled proportion of females was 77.2% (95% CI: 71.2, 83.2; I^2^ = 95.4%), indicating a predominant female representation consistent with the known epidemiology of thyroid disorders. The pooled mean age was 47.2 years (95% CI: 42.6, 51.8; I^2^ = 99.3%), suggesting a middle-aged population, and the pooled mean BMI was 24.6 kg/m^2^ (95% CI: 23.1, 26.1; I^2^ = 93.8%), reflecting generally normal weight distributions with some variation likely due to metabolic effects of hyperthyroidism.

The subclinical hypothyroidism group showed a pooled proportion of females of 69.5% (95% CI: 60.9, 78.1; I^2^ = 97.7%), also highlighting female predominance. The pooled mean age was 52.2 years (95% CI: 48.3, 56.1; I^2^ = 99.8%), indicating an older demographic compared to hyperthyroid groups, and the pooled mean BMI was 27.0 kg/m^2^ (95% CI: 25.9, 28.1; I^2^ = 95.1%), suggestive of a slightly overweight profile that may relate to the subtle metabolic slowdown in this condition.

For the overt hypothyroidism group, the pooled proportion of females was 82.1% (95% CI: 74.4, 89.8; I^2^ = 94.6%), reinforcing the higher prevalence among women. The pooled mean age was 48.0 years (95% CI: 38.3, 57.7; I^2^ = 99.7%), spanning a broad range indicative of varying disease onset, and the pooled mean BMI was 29.4 kg/m^2^ (95% CI: 26.6, 32.2; I^2^ = 96.2%), pointing to a tendency toward overweight or obesity, potentially exacerbated by hypothyroid-induced metabolic changes.

The control group had a pooled proportion of females of 57.7% (95% CI: 51.6, 63.8; I^2^ = 99.9%), showing a more balanced gender distribution. The pooled mean age was 50.6 years (95% CI: 48.6, 52.6; I^2^ = 99.9%), consistent with middle-aged populations, and the pooled mean BMI was 26.0 kg/m^2^ (95% CI: 25.3, 26.7; I^2^ = 99.3%), aligning with normal to slightly overweight ranges in general populations.

### 3.2. Pooled TSH Concentrations Across Thyroid Conditions

The meta-analysis integrated TSH concentration data from 39 studies, encompassing a total of 138,741 observations, to evaluate pooled estimates across four distinct thyroid function categories: overt hyperthyroidism, subclinical hypothyroidism, overt hypothyroidism, and control. Employing a random-effects model, analyses were stratified by group to reflect the diverse TSH profiles associated with each condition. The results revealed significant heterogeneity within all groups (I^2^ ≥ 93.9%), with a highly significant test for subgroup differences (χ^2^ = 521.54, df = 3, *p* < 0.001), indicating distinct TSH distributions across the cohorts.

**Figure 1:**
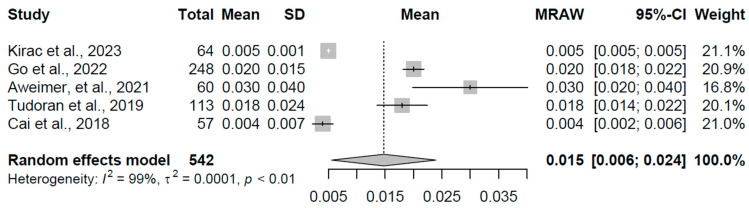
Forest plot of pooled TSH concentrations (mIU/L) for the overt hyperthyroidism group using a random-effects model [16,17,19,20,22].

For the overt hyperthyroidism group, comprising five studies with a combined sample size of 542 participants, the pooled mean TSH concentration was 0.015 mIU/L (95% CI: 0.006, 0.024). Heterogeneity was pronounced (I^2^ = 98.7%, τ^2^ = 0.0001, *p* < 0.001), with individual study estimates ranging from 0.004 to 0.030 mIU/L, suggesting variability potentially linked to differences in disease severity or measurement precision. This is depicted in Figure 1, with a tailored *x*-axis scale from 0 to 0.05 mIU/L to accommodate the low TSH range.

**Figure 2:**
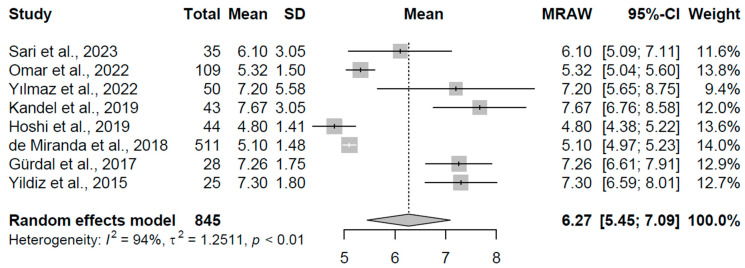
Forest plot of pooled TSH concentrations (mIU/L) for the subclinical hypothyroidism group using a random-effects model [23,24,25,26,27,28,29,30].

In the subclinical hypothyroidism group, data from eight studies with 845 participants yielded a pooled mean TSH of 6.270 mIU/L (95% CI: 5.448, 7.092). Heterogeneity remained substantial (I^2^ = 93.9%, τ^2^ = 1.25, *p* < 0.001), with study-specific means spanning 4.80 to 7.67 mIU/L, indicating a moderate elevation potentially influenced by population characteristics or assay methodologies. The results are illustrated in Figure 2, with an *x*-axis scale from 0 to 10 mIU/L.

**Figure 3:**
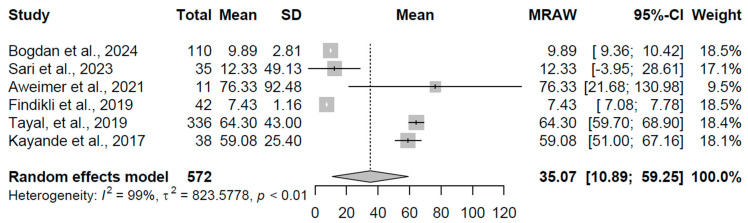
Forest plot of pooled TSH concentrations (mIU/L) for the overt hypothyroidism group using a random-effects model [19,21,23,31,32,33].

The overt hypothyroidism group, based on six studies with 572 participants, exhibited a pooled mean TSH of 35.067 mIU/L (95% CI: 10.89, 59.25), accompanied by extreme heterogeneity (I^2^ = 99.4%, τ^2^ = 823.58, *p* < 0.001). Individual estimates varied widely from 7.43 to 76.33 mIU/L, with the broad confidence interval reflecting this variability. This is visualized in Figure 3, with an *x*-axis scale from 0 to 100 mIU/L to capture the wide range.

**Figure 4:**
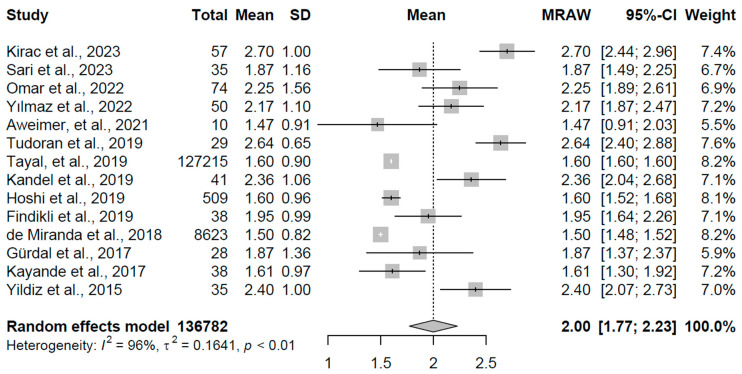
Forest plot of pooled TSH concentrations (mIU/L) for the control group using a random-effects model [16,19,20,21,23,24,25,26,27,28,29,30,32,33].

For the control group, comprising 14 studies with a total of 136,782 participants, the pooled mean TSH was 2.000 mIU/L (95% CI: 1.77, 2.23), with significant heterogeneity (I^2^ = 96.2%, τ^2^ = 0.1641, *p* < 0.001). Study means ranged from 1.47 to 2.70 mIU/L, aligning with euthyroid norms. The findings are presented in Figure 4, with an *x*-axis scale from 0 to 3 mIU/L.

### 3.3. Pooled FT4 Concentrations Across Thyroid Conditions

The meta-analysis synthesized FT4 concentration data from 20 studies, encompassing a total of 158,021 observations, to evaluate pooled estimates across four distinct thyroid function groups. Heterogeneity was evident within most groups, with the magnitude and pattern of FT4 concentrations providing insights into the physiological states of thyroid dysfunction.

**Figure 5:**
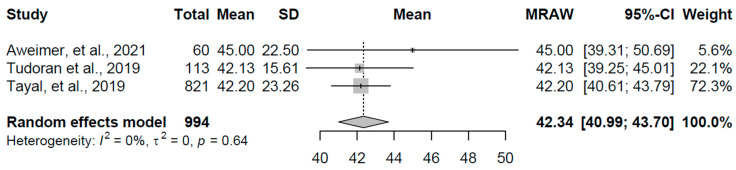
Forest plot of pooled FT4 concentrations (pmol/L) for the overt hyperthyroidism group using a random-effects model [19,20,21].

For the overt hyperthyroidism group, data from three studies with a combined sample size of 994 participants yielded a pooled mean FT4 concentration of 42.343 pmol/L (95% CI: 40.99, 43.70). Heterogeneity was notably low (I^2^ = 0.0%, τ^2^ = 0, *p* = 0.641), suggesting consistency across the included studies, with individual means ranging from 42.13 to 45.00 pmol/L (refer to Figure 5 for forest plot). This elevated FT4 level is consistent with excessive thyroid hormone production, a hallmark of overt hyperthyroidism, and reflects the expected suppression of TSH in this condition, as observed in prior analyses.

**Figure 6:**
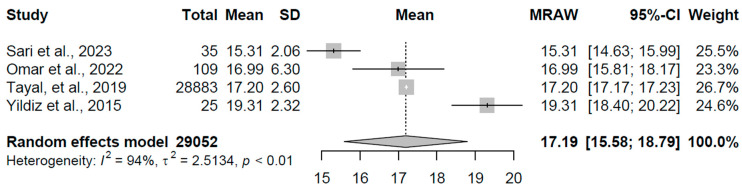
Forest plot of pooled FT4 concentrations (pmol/L) for the subclinical hypothyroidism group using a random-effects model [21,23,24,30].

In the subclinical hypothyroidism group, comprising four studies with 29,052 participants, the pooled mean FT4 was 17.189 pmol/L (95% CI: 15.58, 18.79). Substantial heterogeneity was present (I^2^ = 94.0%, τ^2^ = 2.51, *p* < 0.001), with study-specific means varying from 15.31 to 19.31 pmol/L (refer to Figure 6 for forest plot). This range indicates FT4 levels within or slightly below the normal range, aligning with the definition of subclinical hypothyroidism where thyroid hormone output is insufficient to fully compensate for elevated TSH, potentially reflecting early thyroid gland dysfunction or individual variability in hormone metabolism.

**Figure 7:**
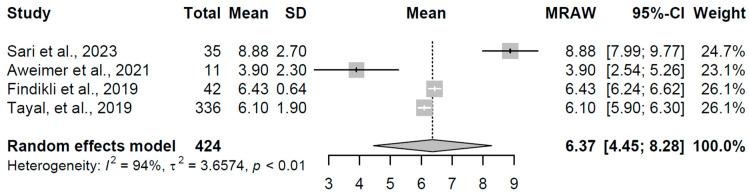
Forest plot of pooled FT4 concentrations (pmol/L) for the overt hypothyroidism group using a random-effects model [19,21,23,32].

The overt hypothyroidism group, based on four studies with 424 participants, exhibited a pooled mean FT4 of 6.365 pmol/L (95% CI: 4.45, 8.28). High heterogeneity was observed (I^2^ = 93.9%, τ^2^ = 3.66, *p* < 0.001), with individual estimates ranging from 3.900 to 8.88 pmol/L (refer to Figure 7 for forest plot). This reduced FT4 concentration is indicative of significant thyroid hormone deficiency, consistent with overt hypothyroidism, where the gland’s inability to produce adequate hormones leads to a compensatory TSH rise. The wide confidence interval suggests variability that may stem from differences in disease severity or treatment status across studies.

**Figure 8:**
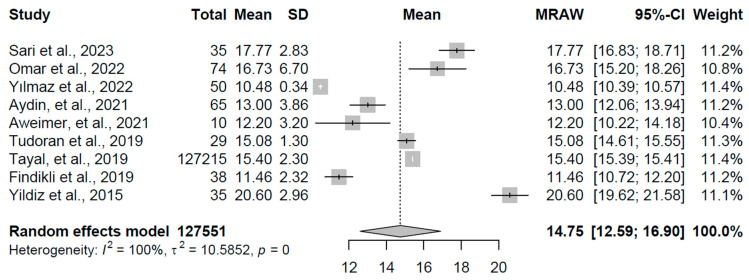
Forest plot of pooled FT4 concentrations (pmol/L) for the control group using a random-effects model [18,19,20,21,23,24,25,30,32].

For the control group, encompassing nine studies with a total of 127,551 participants, the pooled mean FT4 was 14.746 pmol/L (95% CI: 12.59, 16.90). Extreme heterogeneity was evident (I^2^ = 99.9%, τ^2^ = 10.59, *p* < 0.001), with study means spanning 10.48 to 20.60 pmol/L (refer to Figure 8 for forest plot). This range encompasses typical euthyroid FT4 levels, serving as a reference for normal thyroid function, though the pronounced heterogeneity may reflect diverse population characteristics or assay differences among the included studies.

### 3.4. Pooled QTc Interval Durations Across Thyroid Conditions

The meta-analysis synthesized QTc interval duration data from 19 studies, encompassing a total of 31,946 observations, to evaluate pooled estimates across four distinct thyroid function groups. Heterogeneity was substantial within most groups, with the extent and direction of QTc changes providing insights into the cardiac electrophysiological impact of thyroid dysfunction.

**Figure 9:**
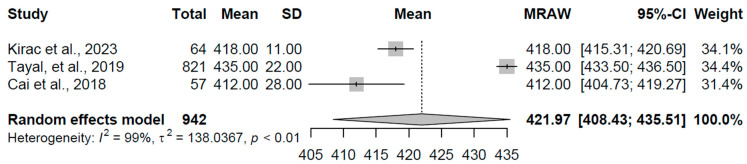
Forest plot of pooled QTc interval durations (ms) for the overt hyperthyroidism group using a random-effects model [16,21,22].

For the overt hyperthyroidism group (k = 3 studies, N = 942), the pooled mean QTc interval duration was 421.97 ms (95% CI: 408.44, 435.51). Pronounced heterogeneity was evident (I^2^ = 98.6%, τ^2^ = 138.04, *p* < 0.001), with individual study means ranging from 412.000 to 435.00 ms (refer to Figure 9 for forest plot). This prolongation aligns with the expected effects of excess thyroid hormones on cardiac repolarization, potentially contributing to increased arrhythmic susceptibility in this condition.

**Figure 10:**
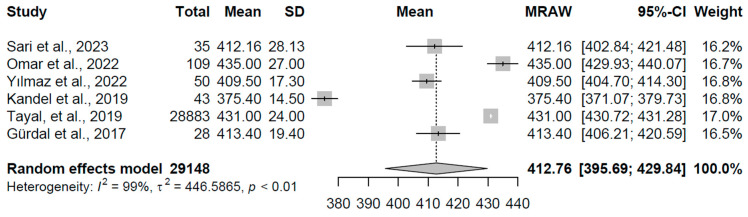
Forest plot of pooled QTc interval durations (ms) for the subclinical hypothyroidism group using a random-effects model [21,23,24,25,26,29].

In the subclinical hypothyroidism group (k = 6 studies, N = 29,148), the pooled mean QTc was 412.77 ms (95% CI: 395.69, 429.84). High heterogeneity was observed (I^2^ = 99.3%, τ^2^ = 446.59, *p* < 0.0001), with study-specific means varying from 375.40 to 435.00 ms (refer to Figure 10 for forest plot). This range suggests a mild to moderate prolongation in some cases, consistent with subtle thyroid hormone deficiencies affecting myocardial conduction, though the broad interval reflects potential influences from comorbidities or measurement variations.

**Figure 11:**
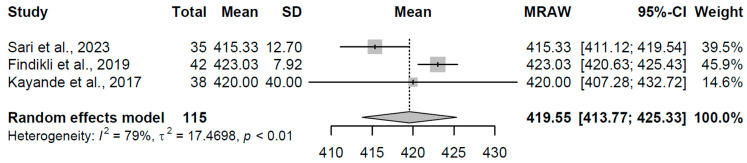
Forest plot of pooled QTc interval durations (ms) for the overt hypothyroidism group using a random-effects model [23,32,33].

The overt hypothyroidism group (k = 3 studies, N = 115) yielded a pooled mean QTc of 419.55 ms (95% CI: 413.77, 425.33). Moderate heterogeneity was noted (I^2^ = 79.5%, τ^2^ = 17.4698, *p* = 0.008), with individual estimates ranging from 415.33 to 423.03 ms (refer to Figure 11 for forest plot). This slight elevation indicates delayed repolarization, a pattern linked to reduced thyroid hormone availability impairing ion channel function in cardiac tissue.

**Figure 12:**
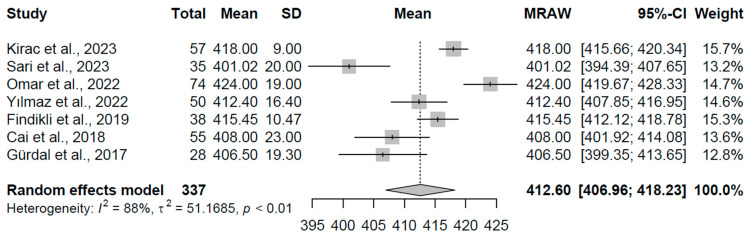
Forest plot of pooled QTc interval durations (ms) for the control group using a random-effects model [16,22,23,24,25,29,32].

For the control group (k = 7 studies, N = 337), the pooled mean QTc was 412.60 ms (95% CI: 406.97, 418.24). Considerable heterogeneity was present (I^2^ = 88.3%, τ^2^ = 51.17, *p* < 0.001), with study means spanning 401.02 to 424.00 ms (refer to Figure 12 for forest plot). This range falls within typical euthyroid QTc norms, serving as a reference and highlighting the relative stability in repolarization-duration-absent thyroid dysfunction, though heterogeneity may arise from demographic or methodological differences among studies.

These pooled QTc estimates are congruent with clinical observations of thyroid hormone’s influence on cardiac electrophysiology. The prolongation in overt hyperthyroidism may stem from enhanced sympathetic tone and ion channel remodeling, while in hypothyroidism, reduced hormone levels could slow repolarization kinetics, potentially elevating risks for arrhythmias such as torsades de pointes. The heterogeneity observed, particularly in subclinical and overt hypothyroidism groups, likely reflects variations in disease stage or concomitant factors.

### 3.5. Pooled Tp-e Interval Durations Across Thyroid Conditions

The meta-analysis synthesized Tp-e interval duration data from 14 studies, encompassing a total of 573 observations, to evaluate pooled estimates across four distinct thyroid function groups: overt hyperthyroidism, subclinical hypothyroidism, overt hypothyroidism, and control. Employing a random-effects model, analyses were stratified by group to reflect the unique Tp-e profiles associated with each condition, revealing notable variations in dispersion durations across the cohorts. Heterogeneity was substantial within most groups, with the extent and direction of Tp-e changes providing insights into the cardiac repolarization impact of thyroid dysfunction.

**Figure 13:**
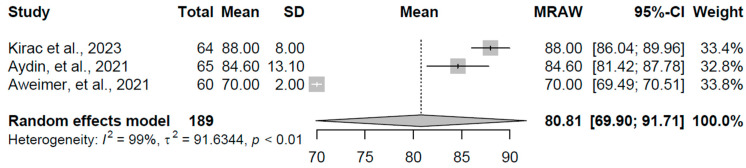
Forest plot of pooled Tp-e interval durations (ms) for the overt hyperthyroidism group using a random-effects model [16,18,19].

For the overt hyperthyroidism group (k = 3 studies, N = 189), the pooled mean Tp-e interval duration was 80.808 ms (95% CI: 69.90, 91.71). Pronounced heterogeneity was evident (I^2^ = 99.5%, τ^2^ = 91.63, *p* < 0.001), with individual study means ranging from 70.00 to 88.00 ms (refer to Figure 13 for forest plot). This prolongation aligns with the expected effects of excess thyroid hormones on ventricular repolarization heterogeneity, potentially contributing to increased arrhythmic vulnerability in this condition.

**Figure 14:**
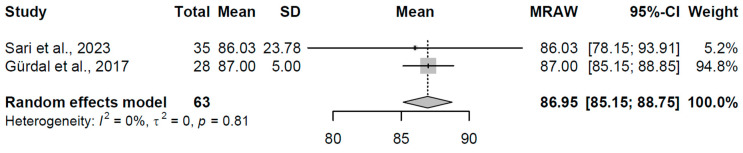
Forest plot of pooled Tp-e interval durations (ms) for the subclinical hypothyroidism group using a random-effects model [23,29].

In the subclinical hypothyroidism group (k = 2 studies, N = 63), the pooled mean Tp-e was 86.95 ms (95% CI: 85.15, 88.75). No heterogeneity was detected (I^2^ = 0.0%, τ^2^ = 0, *p* = 0.81), with study-specific means of 86.03 and 87.00 ms (refer to Figure 14 for forest plot). This consistency suggests stable Tp-e durations in subclinical states, reflecting minimal disruption to repolarization dispersion where thyroid hormone levels remain near normal.

**Figure 15:**
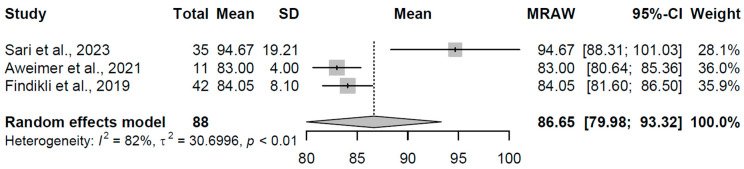
Forest plot of pooled Tp-e interval durations (ms) for the overt hypothyroidism group using a random-effects model [19,23,32].

The overt hypothyroidism group (k = 3 studies, N = 88) yielded a pooled mean Tp-e of 86.654 ms (95% CI: 79.98, 93.32). Moderate heterogeneity was noted (I^2^ = 82.5%, τ^2^ = 30.70, *p* = 0.0033), with individual estimates ranging from 83.00 to 94.67 ms (refer to Figure 15 for forest plot). This slight elevation indicates altered repolarization dynamics, a pattern associated with reduced thyroid hormone availability affecting myocardial ion channel function.

**Figure 16:**
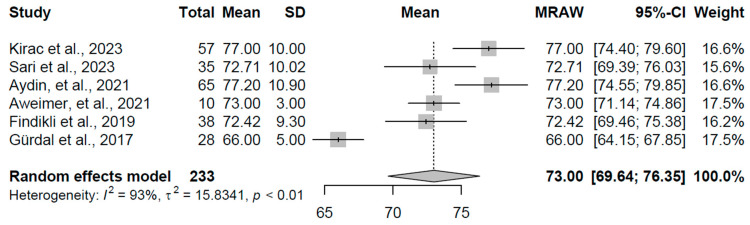
Forest plot of pooled Tp-e interval durations (ms) for the control group using a random-effects model [16,18,19,23,29,32].

For the control group (k = 6 studies, N = 233), the pooled mean Tp-e was 73.00 ms (95% CI: 69.65, 76.35). Considerable heterogeneity was present (I^2^ = 92.9%, τ^2^ = 15.83, *p* < 0.001), with study means spanning 66.00 to 77.20 ms (refer to Figure 16 for forest plot). This range falls within typical euthyroid Tp-e norms, serving as a reference and highlighting the relative stability in repolarization dispersion absent thyroid dysfunction, though heterogeneity may arise from demographic or methodological differences among the included studies.

These pooled Tp-e estimates are congruent with clinical observations of thyroid hormone’s influence on ventricular repolarization. The prolongation in overt hyperthyroidism may stem from heightened myocardial dispersion due to sympathetic overactivation, while in hypothyroidism, diminished hormone levels could impair ion channel kinetics, potentially elevating risks for ventricular arrhythmias. The heterogeneity observed, particularly in overt hyperthyroidism and control groups, likely reflects variations in disease stage or concomitant factors.

### 3.6. Narrative Synthesis

#### 3.6.1. Risk of Arrhythmias

Abnormal thyroid hormone levels have a significant impact on the electrical activity of the heart. In a study conducted on a group of 273 patients with thyroid dysfunction, ECG abnormalities were noted in 67.8% of individuals. Among all recorded abnormalities, arrhythmias predominated, being present in 56% of patients. The proportion of nodal-origin arrhythmias was 25.3%, with the most common being supraventricular tachycardia (15.4%), followed by atrial fibrillation (11.7%) and sinus bradycardia (7.7%). Considering demographic variables, it was shown that the frequency of ECG abnormalities was significantly higher in patients aged 41–60 years. Additionally, it was found that female sex was associated with more than twice the risk of ECG abnormalities compared to males [34].

Similar results were obtained in the study by Mersha BH et al., which analyzed the records of 318 patients with CHyper—ECG was performed in 264 of them, and pathological changes were noted in 52.7% of cases. As in the previous study, supraventricular tachycardia was the most frequently observed. The frequency of symptoms suggesting cardiovascular involvement such as palpitations, dyspnea, chronic fatigue, peripheral edema, and chest pain was 81.1%, 55.3%, 77.4%, 17.6%, and 2.8%, respectively [35].

In another study conducted on 103 patients with primary hyperthyroidism, sinus tachycardia was present in as many as 60.19% of patients, regardless of age group [36].

On the other hand, some studies have indicated that although tachycardia is commonly associated with overt hyperthyroidism, a sinus rhythm exceeding 100 beats/min was observed in only about 28% of patients with biochemically confirmed disease. In the group of patients with the highest FT4 values, this percentage increased to 38.7%, yet remained lower than the data presented in other studies. The authors suggest that the less frequent occurrence of tachycardia may result from earlier disease diagnosis. Interestingly, a more typical sign of hyperthyroidism in this population turned out to be widened pulse pressure (>50 mmHg), which was observed in 64.1% of patients [17].

It was shown that in patients with SCHyper, heart rate was significantly higher than in the control group. A significant negative correlation between TSH levels and heart rate and repolarization markers was also found, suggesting that low TSH may promote tachycardia [37]. In contrast, the study conducted by Rajão et al. did not confirm a significant association between subclinical thyroid dysfunction and the incidence of arrhythmias, conduction disorders, or changes in heart rate on ECG [38]. Moreover, no correlation was found between TSH or FT4 levels and heart rate in any of the groups. The only exception was a weak association between TSH and heart rate in patients with subclinical hypothyroidism. The only statistically significant difference observed was a lower prevalence of bradycardia in patients with subclinical hyperthyroidism [38].

Among patients with overt hypothyroidism, sinus bradycardia was observed in about 15,29% of cases [39]. The incidence of bradycardia was assessed based on the type of thyroid dysfunction in a population without diagnosed arrhythmias. It was demonstrated that in women with CH, heart rate was significantly lower than in the control group. This relationship did not reach statistical significance in men. No significant difference in heart rate was found in patients with SCH, regardless of sex [21].

Yildiz C et al. assessed the presence of heart rate turbulence (HRT) disturbances in patients with SCH. They demonstrated that the values of Turbulence Onset (TO) were higher, and Turbulence Slope (TS) was lower than in the control group, indicating weakened autonomic response. A positive correlation between TSH concentration and the severity of turbulence disturbances was also observed [20,30].

Due to the large number of studies evaluating the impact of thyroid hormones on heart rate variability (HRV), data on this issue are presented collectively in Table 3.

A considerable incidence of conduction disorders was also noted, with a total frequency of 9.5%. Of these, 2.7% referred to AV blocks (1.8% for first-degree AV block and 0.7% for second-degree AV block), 1.8% for right bundle branch block (RBBB), and 0.7% for left bundle branch block (LBBB). For anterior (LAHB) and posterior (LPHB) fascicular blocks of the left bundle branch, the incidence was 2.19% and 0.73%, respectively [34]. In patients with overt hypothyroidism, the presence of AV blocks has been estimated at approximately 12.5%. Arrhythmia was observed in 30.92% of the examined patients. Supraventricular arrhythmia predominated, with its incidence estimated by the authors at 23.02%, while ventricular arrhythmias accounted for 6.57% [44]. Another study also found that the frequency of AV blocks in various thyroid disorders was 1.49%, and that of ventricular tachycardia was 0.47% [45]. In the study by Faisal M et al., this association was not confirmed for AV blocks. In their study, third-degree AV block was found in only 1% of patients with thyroid dysfunction. The authors indicated that the lack of a clear relationship between TSH levels and AV blocks justifies the decision to forgo routine TSH measurement in the diagnosis of such disorders [46].

Data from 24 h Holter ECG monitoring demonstrated that the number of premature ventricular contractions (PVCs) was significantly higher in patients with subclinical hypothyroidism than in the control group (mean 13,104 vs. 9286). A moderate positive correlation between TSH level and the number of PVCs was also noted [47].

#### 3.6.2. Tp-e Changes

An analysis of patients with hypothyroidism revealed significant effects on ventricular repolarization indices, including Tp-e interval, Tp-e/QT, and Tp-e/QTc. A total of 105 patients were analyzed, including those with overt hypothyroidism (CH) and subclinical hypothyroidism (SCH). The control group consisted of euthyroid individuals. The study showed that ventricular repolarization parameters were significantly prolonged in patients with hypothyroidism compared to healthy individuals. Moreover, the authors found a positive correlation between TSH concentration and the length of Tp-e, Tp-e/QT, and Tp-e/QTc. An inverse (negative) correlation was observed between FT4 levels and these ECG parameters. The highest values were recorded in the group of patients with primary hypothyroidism, characterized by the lowest FT4 levels [23].

In the study by Aweimer et al., 90 patients were analyzed and divided into four groups: euthyroid individuals (controls), patients with untreated CH, patients with CH treated with L-thyroxine, and those with thyrotoxicosis. In the group with untreated hypothyroidism, a significant prolongation of Tp-e and JT intervals was observed, whereas in patients treated with LT4, these values normalized. These findings indicate a direct effect of thyroid hormone levels on the duration of ventricular repolarization and suggest that hormone replacement therapy may lead to reversible ECG changes [19].

Similar observations indicated that treatment of patients with overt hypothyroidism restores Tp-e and QTc values to normal. This study showed a strong positive correlation between TSH levels and both Tp-e and QTc. Moreover, ROC curve analysis revealed that Tp-e had greater diagnostic value than QTc in detecting hypothyroidism, with a sensitivity of 77% and specificity of 66% [32].

An analysis of 45 patients with SCHyper compared with a control group revealed significant differences in ventricular repolarization parameters. Patients in the study group had significantly higher values of Tp-e, Tp-e/QT, and Tp-e/QTc. Additionally, increased dispersion of PR, QT, and QTc intervals was noted. A negative correlation was also identified between TSH levels and repolarization parameters, suggesting that even mild forms of hyperthyroidism may affect the electrical stability of the heart. The authors suggested that these changes may serve as potential early markers of arrhythmic risk [37].

In another study, 65 patients with overt CHyper before and after thyroid surgery were examined. It was found that before the procedure, the values of Tp-e, Tp-e/QT, and Tp-e/QTc were significantly elevated, while postoperatively they returned to normal. QT and QTc intervals did not differ significantly before and after surgery [18]. Similar conclusions were made by Hepsen et al. Additionally, they observed that FT4 levels were independently associated with elevated Tp-e/QT values, even within the reference range [48]. In line with these findings, in patients with Graves’ disease, the duration of Tp-e and QTc was significantly prolonged and positively correlated with FT3 and FT4 levels [16].

#### 3.6.3. Changes in Ventricular Repolarization Parameters

A clinical study evaluating the prevalence of electrocardiographic abnormalities in patients with thyroid dysfunction, showed that QTc interval changes accounted for 2.5% of all recorded electrocardiographic abnormalities [34].

The study conducted on 112 patients with newly diagnosed hyperthyroidism, analyzed the effect of thyroid hormones on repolarization parameters, including QTc, QTd, QTe, QTp, and normalized indices: QTe/RR and QTp/RR. Participants were divided into three groups: patients with untreated CHyper, a control group, and patients with CHyper after initiation of treatment. In the first group, QTc was slightly prolonged (412 ± 28 ms) compared to both the control group and the treated group. A similar trend was observed for QTd—its highest values were noted in patients before treatment. QTe/RR and QTp/RR parameters were significantly increased in the group with untreated CHyper. No significant differences were observed between the control group and the post-treatment group [22].

In the study by Kayande et al., involving a group of 38 women with overt hypothyroidism, it was found that a three-month levothyroxine therapy significantly improved the hormonal profile (increase in T3 and T4, decrease in TSH), and also led to QTc interval shortening—from 0.42 ± 0.04 s before treatment to 0.40 ± 0.03 s after treatment. A positive correlation between TSH concentration and QTc duration was also observed, suggesting that treatment reduces ventricular repolarization heterogeneity and may lower the risk of ventricular arrhythmias in patients with overt hypothyroidism [33].

In a study conducted on women with SCH, increased QT dispersion was demonstrated compared to women with normal thyroid hormone levels. At the same time, a weak correlation was observed between QTd values and TSH concentration. Moreover, no significant differences were found between the groups in terms of QT and QTc durations [26].

The significant impact of thyroid hormones on QT interval duration was also confirmed in the study by Liu C et al., which showed that QT duration may depend not only on changes in FT3 and FT4 levels but also on TSH, even when thyroid hormone levels remain within the normal range. The study involved 271 patients with thyroid cancer, divided into three groups according to TSH levels: < 0.1 mIU/L, 0.1–0.5 mIU/L, and 0.5–2.0 mIU/L. The longest QTmax and QTcmax values were recorded in the groups with the lowest TSH levels. A similar relationship was observed for QTd and QTcd—an inverse correlation between TSH level and these parameters was demonstrated. Interestingly, even in the group with normal TSH (0.5–2.0 mIU/L), a positive correlation was observed between TSH level and QTd and QTcd values—suggesting that higher TSH, even within the normal range, may be associated with greater QT dispersion [40].

A positive correlation between TSH concentration and QTc duration was also demonstrated in the study conducted on 30 women with SCH. In this group, QTc prolongation was the only significant abnormality—no significant differences were observed compared to the control group in QRS duration, PR interval, or QRS axis [49].

#### 3.6.4. Changes in P-Wave Duration, PQ Interval, QRS Duration and ST-T Morphology

A study conducted in Copenhagen on 132,707 patients demonstrated that individuals with overt hyperthyroidism (CHyper) had a shortening of the P-wave duration and PR interval by an average of 3 ms. Similar changes were observed in subclinical hyperthyroidism (SCHyper), where the PR interval was prolonged by an average of 1.5 ms. In overt hypothyroidism (CH), PR prolongation was more pronounced, averaging 6.6 ms [21].

The effect of thyroid hormones on the QRS complex was assessed, among others, in a study by Sawarthia P et al., which showed that the most common abnormality in patients with CH was reduced QRS voltage—observed in 30.9% of cases. In the same group, prolonged QRS duration was found in 2.9% of patients. This study also showed that RBBB occurred in 7.4% of patients with CH [50]. Ohal et al. reported similar findings, observing low QRS voltage in 26.67% of patients [51]. In the study involving 50 patients with CH, the most frequently observed change was also low QRS voltage—present in 66% of cases. The second most common abnormality was nonspecific ST–T changes, detected in 8% of patients [52]. Poor R-wave progression was noted in 11% of patients with overt hypothyroidism [39].

Another study demonstrated that fragmented QRS (fQRS) complexes were significantly more frequent in patients with SCH. The authors also found a correlation between the presence of fQRS and left ventricular dysfunction—both systolic and diastolic—assessed via the Myocardial Performance Index (MPI). It was noted that elevated MPI values may serve as an independent marker of cardiovascular risk, including increased mortality, regardless of classical risk factors [25].

Among 103 patients with overt hyperthyroidism, deviation of the electrical axis of the heart was detected in 8.8% [34]. Another study confirmed the shortening of QRS duration in patients with CHyper [21].

The study involving 273 patients with both CH and CHyper, showed that the overall prevalence of ST-segment abnormalities reached 15%, while T-wave abnormalities were present in 9.5% of cases [34].

Finally, Guan F et al. analyzed a group of 260 patients who had undergone thyroidectomy. In a subset of these patients, levothyroxine was intentionally withdrawn for three weeks. In this group, significant ECG changes were observed, the most frequent being T-wave flattening or inversion in the inferior leads. A positive correlation was found between the severity of these changes and serum TSH levels—both the average value and the level measured at the end of the levothyroxine withdrawal period [53].

### 3.7. Study Quality Assessment

Overall, the methodological quality of the included studies was moderate to good. Most studies scored well in the selection and outcome domains, whereas comparability was the most common source of bias due to limited control for confounders. No study was excluded based on low quality, but NOS ratings were considered during the interpretation of the pooled results.

## 4. Discussion

In this systematic review and meta-analysis, we found that thyroid dysfunction is consistently associated with alterations in both thyroid hormone levels and ECG repolarization markers. Importantly, we observed prolonged QTc and Tp-e intervals in overt hyperthyroidism and overt hypothyroidism compared with euthyroid controls, suggesting increased heterogeneity of ventricular repolarization across the spectrum of thyroid dysfunction.

TH exhibit positive inotropic and chronotropic effects, increase ejection fraction and cardiac output. At the same time, they lower peripheral vascular resistance by stimulating nitric oxide synthesis by endothelial cells, leading to vasodilation. Reduced vascular resistance results in decreased renal perfusion and secondary activation of the renin–angiotensin–aldosterone system, which may exacerbate cardiovascular dysfunction [12,54]. The effects of thyroid hormones also include an indirect impact on lipid metabolism—through the activation of the enzyme 7α-hydroxylase, leading to cholesterol degradation and transformation of low-density lipoproteins (LDLs) [12]. They also support the process of angiogenesis [54]. Abnormal thyroid hormone levels may lead to the development of arterial hypertension. In hyperthyroidism, elevated systolic blood pressure is more commonly observed, whereas in hypothyroidism—an increase in diastolic or mean pressure is more typical [55].

To better understand the discussed thyroid disorders, the hormonal characteristics are presented in Table 4.

ECG is a fast, simple, and highly effective diagnostic tool commonly used as a screening test in conditions such as myocardial infarction, myocardial hypertrophy, ischemic heart disease, as well as for detecting and assessing the risk of various types of arrhythmias [48,57]. As it turns out, thyroid dysfunctions are also reflected in ECG recordings. Moreover, detected electrocardiographic changes in patients with thyroid disorders may serve as an early indicator of the risk of developing cardiac arrhythmias or thromboembolic events, such as ischemic stroke.

To better understand the analyzed ECG parameters, their definitions and a brief description are presented in Table 5.

Importantly, the electrophysiological markers demonstrated that both overt hyperthyroidism and overt hypothyroidism were associated with clear abnormalities in ventricular repolarization. QTc intervals were prolonged in both conditions compared with euthyroid controls, with mean QTc values reaching approximately 422 ms in overt hyperthyroidism and 420 ms in overt hypothyroidism, versus 413 ms in controls. Similarly, Tp-e intervals—reflecting transmural dispersion of repolarization—were elevated in these groups, with pooled means around 81 ms in overt hyperthyroidism and 87 ms in overt hypothyroidism, compared with 73 ms in controls. In contrast, subclinical hypothyroidism demonstrated only mild biochemical abnormalities and correspondingly limited ECG alterations. QTc prolongation in this group was modest, and Tp-e values remained relatively preserved, suggesting that subtle thyroid hormone imbalances may have a less pronounced impact on myocardial repolarization dynamics. Taken together, these results imply that clinically overt thyroid dysfunction exerts the most significant influence on ECG markers of arrhythmic risk, whereas subclinical disease may represent an intermediate phenotype with smaller electrophysiological consequences.

The duration of the Tp-e interval is important in assessing the risk of arrhythmias. Prolongation of Tp-e and its parameters—Tp-e/QT and Tp-e/QTc—is positively correlated with the risk of ventricular arrhythmias and increases the risk of cardiovascular mortality [58]. The prolongation of these parameters reflects abnormal repolarization occurring between the epicardium and endocardium, which predisposes cardiac arrhythmias [71]. As demonstrated by a meta-analysis conducted by Tse G et al., the risk of ventricular arrhythmias and death in patients with prolonged Tp-e is increased approximately 1.59-fold in the general population [72]. It should be emphasized that Tp-e/QT and Tp-e/QTc are more sensitive markers than Tp-e alone, QT dispersion (QTd), or QTc dispersion (QTcd), due to the fact that they are independent of heart rate [59,60,73]. The prolongation of the above-mentioned markers occurs, among others, in long QT syndrome (LQTS), Brugada syndrome, Fabry disease, acute myocarditis, arrhythmogenic right ventricular cardiomyopathy, and in patients with acromegaly [58]. As demonstrated in our systematic review, these parameters are also prolonged in conditions associated with reduced levels of free thyroid hormones in the serum, whereas they are shortened in diseases characterized by excessively low levels of TH.

The QT interval is a kind of parameter of electrical stability [61]. Prolongation of QT (LQTS) may be caused by many clinical situations—medications used, electrolyte disturbances, presence of comorbidities, arrhythmias, structural heart disease, or genetic mutations of ion channels [74,75]. Our analysis also showed that LQTS occurs in individuals with elevated levels of thyroid hormones, which is associated with an increased arrhythmogenic risk and the occurrence of life-threatening ventricular arrhythmias, including polymorphic ventricular tachycardia and sudden cardiac death (SCD). In atypical cases, it is also associated with atrioventricular blocks (AV blocks), sinus bradycardia, and atrial-origin arrhythmias [76,77]. QT shortening, like LQTS, can also lead to life-threatening ventricular and supraventricular arrhythmias, as well as cardiac arrest. A shortened QT interval may result from genetic mutations of ion channels, referred to as short QT syndrome (SQTS), as well as from secondary causes—including electrolyte disturbances, the action of certain medications, and, as our review has shown, thyroid dysfunction [78,79]. Similar associations have been demonstrated for QTc, QTd, and QTcd [76,77,80,81].

From a clinical perspective, our results support routine consideration of ECG repolarization markers in patients with overt thyroid dysfunction, particularly in those with additional proarrhythmic risk factors. Prolonged QTc and Tp-e intervals may help identify individuals at higher risk of ventricular arrhythmias, especially during periods of unstable thyroid function or rapid dose adjustments. In subclinical hypothyroidism, the relatively preserved repolarization parameters observed in our analysis suggest that ECG-based risk stratification may be less informative, although selected high-risk patients might still benefit from closer monitoring.

As we have shown, TH also influence the duration of the P wave on ECG; their excess leads to a shortening of the P wave duration [21]. It has been demonstrated that changes in P wave duration beyond the reference range are associated with an increased risk of atrial fibrillation (AF) [82,83]. The mechanism of AF development based on P wave shortening is explained by increased atrial conduction velocity. As a result, AF may develop in up to 20% of patients [82]. Similarly, prolongation of P wave duration predisposes to AF and increased risk of SCD [83]. P wave dispersion can also be used to predict the occurrence of AF. The correlation between changes in TH levels and changes in Pd on ECG and the risk of AF reflects the previously mentioned correlation between P wave duration and AF occurrence [66].

Not only the P wave but also the PR segment shows abnormalities in individuals with thyroid dysfunction. Thyroid-related disorders lead to its prolongation [68]. A meta-analysis conducted by Kwok CS et al. suggest that an increase in PR interval is associated with the risk of heart failure development, AF, and increased mortality [67]. The authors also mention the possibility of progression from first-degree AV block, characterized by PR interval prolongation, to higher, more advanced blocks.

Thyroid hormone level changes significantly affect the duration and morphology of the QRS complex. In hypothyroidism, the QRS complex duration is shortened, whereas in hyperthyroidism, QRS prolongation is observed [21,50]. QRS prolongation has been associated with an increased frequency of hospitalizations due to heart failure, worsening of related symptoms, increased heart rate, higher baseline NT-proBNP, and is positively correlated with impaired left ventricular function [68]. Increased presence of fQRS observed in thyroid disorders correlates with impaired left ventricular function, increases the risk of ventricular arrhythmias and SCD, and is an independent predictor of mortality [69].

Changes in the T wave occur in many disease entities, including thyroid dysfunction [53,84,85]. T wave inversion is visible, among others, in acute coronary syndromes, myocardial infarction, hypertrophic cardiomyopathy, neurological diseases, as a result of certain medications, and in pulmonary embolism. Changes in T wave morphology may also be a variant of normal; therefore, clinical context and comparative analysis with previous ECGs may be invaluable in assessing the clinical significance of T wave changes. However, careful analysis of this part of the electrocardiogram may lead to many important clinical insights and often reduce the use of unnecessary procedures and tests [84,85].

Thyroid diseases are also associated with numerous cardiac rhythm disturbances. Due to increased sympathetic activity and reduced vagal tone, there is a decrease in heart rate variability (HRV), which predisposes to supraventricular arrhythmias, including atrial fibrillation [86]. Reduced HRV values also serve as a marker of cardiovascular risk [70]. This mechanism occurs not only in overt, fully symptomatic forms of the disease but also in its subclinical states, suggesting that HRV depends not only on free TH levels but also on TSH. Reduced vagal tone predisposes to increased mortality in patients without organic heart disease [86].

AF is the second most common cardiac arrhythmia, right after supraventricular tachycardia. Its frequency is positively correlated with age. In thyroid disorders, it usually presents as persistent AF [87]. The presence of AF leads to an increased risk of stroke and heart failure [88]. Patients with AF have a 3.67-fold higher all-cause mortality risk compared to the general population and over 5.5-fold higher risk due to cardiovascular causes [89]. AF is the main trigger of cardioembolic events and accounts for 15% to 24% of ischemic strokes. Stroke resulting from AF is associated with a higher risk of death, a more severe course, and an increased rate of recurrence [90]. It should also be mentioned that high levels of thyroid hormones are associated with coagulation disorders, which may be an independent risk factor for stroke [88]. Proarrhythmic effects are also mediated by increased activity and automaticity of cardiomyocytes located in the pulmonary veins, which can directly cause AF [13].

Thyroid hormone receptors are also found within fibroblasts located in the heart muscle. It has been shown that T3 deficiency leads to increased fibroblast proliferation and increased DNA and mRNA synthesis of collagen. On the other hand, TH supplementation increases the activity of metalloproteinases, which limit the process of myocardial fibrosis. Myocardial fibrosis is directly associated with an increased risk of AF. Another mechanism by which AF develops in the context of thyroid hormones is the induction of oxidative and mitochondrial stress, as well as inflammation caused by excess TH [11].

Considering the high prevalence and clinical importance of early AF detection, screening of free thyroid hormones and TSH is extremely important. From 55% to 75% of patients with AF can be cured within 3–6 months by normalizing free TH levels [87].

Several potential confounders may influence the relationship between thyroid hormone levels and ECG parameters. These include the use of medications affecting cardiac conduction or repolarization (e.g., β-blockers, antiarrhythmic drugs), electrolyte disturbances (particularly potassium, calcium, and magnesium), and coexisting cardiovascular conditions such as ischemic heart disease, heart failure, or structural abnormalities. As the included studies varied in how these factors were reported or controlled for, some residual confounding cannot be fully excluded.

From a practical clinical perspective, our findings highlight that simple ECG-derived repolarization markers such as QTc and Tp-e may provide a readily accessible tool to identify patients with overt thyroid dysfunction who may exhibit increased ventricular electrical instability.

The risk-of-bias assessment indicated generally moderate methodological quality, with some variability in population selection and adjustment for confounders. Although these limitations may partly explain the heterogeneity observed in the pooled ECG parameters, the overall direction of the effects remained consistent across studies.

## 5. Limitations

The present review is subject to several limitations. First, the included studies demonstrated considerable heterogeneity in terms of design, population size, and classification of thyroid dysfunction, which may limit the comparability and generalizability of the findings. Second, most studies were cross-sectional in nature, making it difficult to establish causal relationships or evaluate the long-term effects of thyroid hormone fluctuations on electrocardiographic parameters. Third, inconsistencies in ECG methodology—such as differences in measurement techniques (manual versus automated), lead selection, and QTc correction formulas—may introduce measurement bias and contribute to data heterogeneity. Although a formal risk of bias assessment was not performed, the included studies were qualitatively evaluated for methodological transparency, population selection, and completeness of ECG and hormonal data. Overall, their methodological quality was acceptable, though the observational design and variable reporting standards may have introduced a moderate risk of bias. An additional limitation concerns the lack of standardization in ECG methodology across included studies. Different correction formulas for QTc (e.g., Bazett vs. Fridericia), manual versus automated measurement techniques, and variability in the number and type of analyzed leads may have introduced bias and contributed to the observed heterogeneity. These methodological inconsistencies reduce comparability and should be addressed in future research through standardized protocols.

## 6. Conclusions and Future Directions

This systematic review confirms that thyroid dysfunction is associated with significant alterations in ECG parameters. Incorporating ECG into the diagnostic and monitoring process of thyroid disease may improve cardiovascular risk stratification. However, evidence is insufficient to recommend its routine use as a universal screening tool. Future prospective, large-scale cohort studies are needed to determine the clinical utility of standardized ECG assessment in this population. Future research should aim to address several important gaps highlighted by this systematic review and meta-analysis. First, standardized protocols for ECG acquisition and repolarization measurements (including QTc and Tp-e) are urgently needed, as methodological inconsistency contributed substantially to the heterogeneity observed across studies. Additionally prospective cohort studies and well-designed longitudinal analyses are required to determine whether the observed QTc and Tp-e prolongation in overt thyroid dysfunction translates into a measurable increase in ventricular arrhythmias, syncope, or sudden cardiac death. Linking electrophysiological markers with real-world clinical endpoints would significantly strengthen risk stratification strategies. Future investigations should explore effect modifiers such as age, sex, autoimmunity, comorbid cardiovascular disease, and the use of medications influencing repolarization. These factors may explain some of the variability in ECG parameters and should be incorporated into subgroup or multivariable analyses. Future studies should also assess the reversibility of QTc and Tp-e abnormalities after restoration of euthyroidism to determine periods of highest arrhythmic vulnerability. In addition, the use of advanced analytic tools, including Holter monitoring and AI-assisted ECG interpretation, to more precisely characterize repolarization abnormalities and refine arrhythmic risk stratification in thyroid dysfunction.

## Figures and Tables

**Table 1:** Search Strategy and Databases Used for the Systematic Review and Meta-Analysis.

Data Base	Keywords Scope	Logical and Restrictive Operators
PubMed	“Electrocardiography”[Mesh], “Electrocardiography”[tiab], “ECG”[tiab], “Heart Conduction System”[Mesh], “Heart Conduction System”[tiab], “Heart Rate”[Mesh], “Heart Rate”[tiab], “heart rate variability”[Mesh], “heart rate variability”[tiab], “Electrocardiographic Changes”[tiab], “QT”[tiab], “QTc”[tiab], “Tp-E interval”[tiab], “Cardiac Repolarization”[tiab], “T wave”[tiab], “QRS”[tiab] OR “P wave”[tiab], “ST-T”[tiab], “PR interval”[tiab], “Thyroid Hormones”[Mesh],”Thyroid Hormones”[tiab], “Thyroxine”[Mesh], “Thyroxine”[tiab], “Thyroid Gland”[Mesh], “Thyroid Gland”[tiab], “Thyroid Dysfunction”[tiab], “Hyperthyroidism”[Mesh], “Hyperthyroidism”[tiab],”Hypothyroidism”[Mesh] “Hypothyroidism”[tiab], “Thyroid Neoplasms”[Mesh] “Thyroid Neoplasms”[tiab], “Thyroid diseases”[Mesh] “Thyroid diseases”[tiab]	AND, OR, NOT, [Mesh], [tiab], “humans”[MeSH Terms], “Adult”[MeSH], NOT “Review”, NOT “Case Reports”
Science Direct	“Tp-e”, “T wave”, “QT”, “QRS”, “P wave”, “ST-T”, “PR interval”, “Heart rate variability”, “hyperthyroidism”, “hypothyroidism”, “thyroid disease”	AND, OR, NOT NOT “case report”, NOT “children”
Google Scholar	“Tp-e”, “T wave”, “QT”, “QRS”, “P wave”, “ST-T” OR “PR interval”, “Heart rate variability”, “hyperthyroidism”, “hypothyroidism”, “thyroid disease”, “Thyroid Dysfunction”, “Thyroid Neoplasms”	AND, OR, -review, -children

**Table 2:** PICO Framework Used for the Systematic Review and Meta-Analysis.

Element	Inclusion Criteria	Exclusion Criteria
P (Population)	Adult patients with thyroid dysfunction	ECG changes not a primary outcome insufficient sample size (<30 patients) acute or critical condition of the study group focused solely on atrial fibrillation full text not available insufficient ECG detail for data extraction
I (Intervention/Exposure)	Abnormal levels of thyroid hormones (TSH, FT3, FT4), suppressive or substitution therapy	
C (Comparison)	Individuals with normal thyroid function (euthyroidism) or varying degrees of thyroid dysfunction	
O (Outcome)	Changes in the electrocardiogram (including alterations in QT and QTc intervals and related parameters, Tp-e duration and related parameters, changes in the morphology and duration of the QRS complex, P wave, PR interval, T wave morphology and prevalence of arrythmias) as well as changes in sinus rhythm variability analysis.	
S (Study design)	Original research articles	

**Table 3:** Characteristics of Studies Evaluating Heart-Rate Variability.

Reference Number	Study Design	Population	Exclusion Criteria	Outcomes
[40]	Retrospective cohort study	Differentiated thyroid cancer	- Major or uncontrolled cardiac disease, - incomplete demographic data, TSH > 2.0 mIU/L, - recent use of other medications (within 3 months), or significant comorbidities (e.g., diabetes, dyslipidemia, heavy smoking or alcohol use).	Patients with suppressed TSH levels, especially had significantly reduced HRV parameters to euthyroid patients. The LF/HF ratio was elevated, indicating autonomic imbalance.
[22]	Observational cross-sectional case–control study	Chyper	- Presence of significant thyroid-related disorders or complications, - major cardiovascular or metabolic diseases, - use of medications affecting cardiac rhythm or thyroid function, - hematologic abnormalities, - poor-quality or insufficient ECG recordings, - substantial arrhythmia burden, - any condition that prevented effective participation in the study.	Hyperthyroid patients exhibited significantly decreased HRV parameters and increased LF/HF ratio, indicating sympathovagal imbalance. After normalization of thyroid hormone levels, all parameters returned to control values.
[20]	Observational cross-sectional case–control study	Chyper	- Presence of subclinical hyperthyroidism, - male participants, - postmenopausal women, - individuals with diabetes, neurological or respiratory disorders, or any significant cardiovascular disease unrelated to thyrotoxicosis, - those with systolic blood pressure above 150 mmHg and/or diastolic pressure above 90 mmHg, - using beta-blockers, - without sinus rhythm, - with fewer than five isolated premature ventricular contractions on 24 h Holter ECG monitoring,	Time-domain HRV parameters were significantly reduced in all patients. In contrast, frequency-domain results were less conclusive: total power was reduced only in severe cases, while LF and HF were elevated but with normal LF/HF ratio. SDNN and HRVTI were positively correlated with TSH and negatively with FT4 levels and disease duration.
[41]	Descriptive cross-sectional observational study	CH	- History of cardiovascular, renal, hepatic, or psychiatric disorders. - Acute or chronic medical condition; diabetes mellitus; alcohol dependence; leprosy; neuromuscular diseases; drug-related neuropathy or myopathy; hypothyroidism	In terms of HRV, all time-domain parameters and key frequency-domain parameters were significantly reduced in hypothyroid patients suggesting diminished parasympathetic modulation. LF/HF ratio and normalized values remained comparable between groups.
[27]	Cross-sectional analysis of the baseline data of ELSA-Brasil	SCH	- Not validated HRV, - lack of information about thyroid function, - medications which can interfere thyroid functions or autonomic modulation	In the resting supine position, SCHypo subjects showed significantly increased 0 V patterns (sympathetic modulation) and decreased 2 V patterns (parasympathetic modulation), indicating autonomic imbalance. No significant differences were detected in standard time or frequency domain indices. After active postural change, SCHypo patients exhibited a blunted autonomic response, especially a reduced increase in the 0 V patter.
[31]	Prospective observational study	CH	- Individuals with previous cardiovascular disease, - medications that could affect thyroid function or autonomic modulation, - chronic obstructive pulmonary disease, liver or renal insufficiency, malignancy.	Hypothyroid patients showed significantly reduced time-domain HRV parameters (SDNN, RMSSD, pNN50) and frequency-domain values (TP, HF), with elevated LF/HF ratio, indicating reduced parasympathetic and increased sympathetic activity. After 3 months of treatment, all HRV parameters improved significantly but differences remained between severe and non-severe hypothyroid subgroups.
[28]	Cross-sectional analysis of the baseline data of ELSA-Brasil	SCHyper, SCH	- Individuals with overt thyroid disease, previous cardiovascular disease, - use of medications that could affect thyroid function or autonomic modulation, - with invalid or poor-quality HRV recordings—thyroid disorders that did not meet the criteria	In unadjusted analyses, the SCHyper group exhibited lower SDNN and LF values compared to controls, indicating reduced HRV and potential autonomic imbalance. However, after full adjustment for confounding variables (age, sex, race, BMI, smoking, physical activity, etc.), these associations lost statistical significance. No significant differences in HRV parameters were observed in the SCHypo group in either unadjusted or adjusted analyses.
[42]	Observational case–control study (cross-sectional)	CH	- comorbid diseases, - thyroid dysfunction other than newly diagnosed hypothyroidism - individuals outside the age range of 20–40 years, or those who did not provide consent were excluded.	The overall comparison between the two groups did not show statistically significant differences in mean R-R interval, SDRR, or RMSSD, a significant reduction in SDRR and RMSSD was observed in the subgroup of hypothyroid patients with TSH > 100 µIU/mL.
[43]	Cross-sectional analysis (included within the prospective, multicentre ELSA-Brasil cohort)	SCH	- previous cardiovascular disease, - thyroid diseases - use of pacemakers, - use of beta-blockers and medication which can affect thyroid function.	While SCH alone did not significantly alter HRV parameters compared to controls, patients with both DM and SCH showed significantly lower values of HRV indices (SDNN, RMSSD, LF, HF). Odds ratios for altered HR and HRV were higher in the DM + SCH group compared to DM alone. However, after adjusting for disease severity markers (HbA1c and TSH), the associations lost statistical significance.

**Table 4:** Patterns of Thyroid Hormone Alterations in Thyroid Diseases. TSH is a tropic hormone secreted by the anterior pituitary gland that regulates thyroid function by stimulating the synthesis and secretion of the hormones T3 and T4, as well as promoting the growth of thyrocytes. Its secretion is subject to negative feedback, which depends on the levels of free thyroid hormones in the blood. FT4 is the main hormone secreted by the thyroid gland, representing the biologically active, protein-unbound fraction of thyroxine. It primarily acts as a prohormone that is converted into FT3 in peripheral tissues. FT3 is the active form of the thyroid hormone, produced mainly through peripheral deiodination of FT4 [56].

Thyroid Diseases	Thyroid Stimulating Hormone (TSH)	Free triiodothyronine (FT3)	Free thyroxine (FT4)
CHyper [56]	↓	↑	↑
SCHyper [56]	↓	Normal	Normal
CH [56]	↑	↓	↓
SCH [56]	↑	Normal	Normal

Note: The arrows indicate the characteristic direction of change of each hormone (TSH, FT3, FT4) in the respective thyroid disorders presented in the table. Upward arrows (↑) denote an increase, while downward arrows (↓) denote a decrease relative to the normal physiological range. These arrows illustrate typical hormonal patterns observed in overt and subclinical hyperthyroidism or hypothyroidism.

**Table 5:** Electrocardiographic Parameters.

ECG Parameters	Definition	Characteristic
Tp-e	T peak to T end; expresses the time between the peak of the T wave and its end [58].	An index defining the transmural difference in repolarization time between different layers of the heart muscle [58].
Tp-e/QT	Ratio of Tp-e duration to total QT interval [58,59,60].	Reflects the relative dispersion of ventricular repolarization [58,59,60].
Tp-e/QTc	Ratio of Tp-e to corrected QT interval [58,59,60].	Reflects the relative dispersion of ventricular repolarization independent of heart rate [58,59,60].
QT	Time measured from the beginning of the Q wave to the end of the T wave [61].	It determines the time during which depolarization and repolarization of the ventricular muscle occurs, i.e., it covers the entire duration of the ventricular action potential [61].
QTc	QT interval corrected for heart rate [62].	Allows the assessment of ventricular repolarization time independently of the heart rate [62].
QTd	The difference between the longest and shortest QT intervals measured in different electrocardiogram leads [63].	It reflects the heterogeneity of ventricular repolarization in space, i.e., the differentiation of repolarization times in different areas of the ventricular muscle [63].
QTe	The interval from the beginning of the Q wave to the end of the T wave [64].	It corresponds to the total time of depolarization and repolarization of the heart ventricles [64].
QTcd	QTd corrected for heart rate [63].	It reflects the spatial heterogeneity of ventricular repolarization independent of heart rate [63].
QTp	The interval measured from the beginning of the QRS complex to the peak of the T wave [65].	It represents the duration of the early phase of ventricular repolarization [65].
Pd	The difference between the longest and shortest P wave duration [66].	It reflects the heterogeneity of atrial conduction and is a noninvasive indicator of atrial electrical instability [66].
PR interval	A fragment of an ECG recording measured from the beginning of the P wave to the beginning of the QRS complex [67,68].	It covers the time of conduction of an electrical impulse from the sinus node to the muscular layer of the heart ventricles [67,68].
fQRS	Presence of additional breaks in a narrow (<120 ms) QRS complex [69].	It demonstrates conduction heterogeneity resulting from myocardial scarring [69].
TO	An indicator of the percentage change in heart rate following a premature ventricular beat [11].	Reflects the early phase of the sinus response [11].
TS	Index of the slope of heart rate recovery following a premature ventricular contraction [11].	Reflects sinus reflex activity and autonomic nervous system function [11].
**Parameters used in the analysis of heart rate variability [70]**		
**Time-domain**		
RR	Time between two consecutive R waves.	The time corresponding to the length of one heart cycle, used to assess the rate
SDNN	Standard deviation of RR intervals	Assessment of global activity of the autonomic nervous system.
RMSSD	Root mean square of successive RR-intervals differences	Reflects mainly parasympathetic activity. Associated with HF power.
pNN50	Percentage of adjacent NN intervals varying by more than 50 milliseconds	A parameter used to assess short-term heart rate variability and parasympathetic activity. Related to HF power.
**Frequency domain**		
TP	Total power of HRV signal	The sum of the energy of all analyzed bands: very low frequency, low frequency, high frequency.
VLF	Very Low Frequency	The lowest frequency band in spectral analysis, covering the range from 0.0033 to 0.04 Hz
LF	Low Frequency	One of the main frequency bands analyzed in the spectral evaluation covering the range from 0.04 to 0.15 Hz.
HF	High Frequency	A frequency band in spectral analysis spanning the range from 0.15 to 0.4 Hz. It is considered the most sensitive and specific indicator of parasympathetic nervous system activity.
LF/HF	LF to HF ratio	Determines sympathovagal balance

## Data Availability

The raw data supporting the conclusions of this article will be made available by the authors on request.

## Supplementary Materials

The following supporting information can be downloaded at: https://www.mdpi.com/article/10.3390/jcm14248755/s1, Table S1. PRISMA Checklist.

## Abbreviations

AF	atrial fibrillation
AV block	atrio-ventricular blocks
CCS	cardiac Conduction System
CH	clinical hypothyroidism
CHyper	clinical hyperthyroidism
CVS	cardiovascular system
ECG	electrocardiography
fQRS	fragmented QRS complex
FT3	free triiodothyronine
FT4	free thyroxine
HR	heart rate
HRT	heart Rate Turbulence
HRV	heart rate Variability
LAHB	left anterior fascicular block
LBBB	left bundle branch block
LDL	low density lipoprotein
LPHB	left posterior fascicular block
LQTS	long QT syndrome
MPI	myocardial performance index
PRISMA	Preferred Reporting Items For Systematic Reviews And Meta-Analyses
PVC	premature ventricular contraction
QTcd	QTc dispersion
QTd	QT dispersion
RBBB	right bundle branch block
RHR	resting heart rate
SCD	sudden cardiac death
SCH	subclinical hypothyroidism
SCHyper	subclinical hyperthyroidism
SQTS	short QT syndrome
TH	thyroid hormones
TO	turbulence onset
TRs	thyroid receptors
TS	turbulence Slope
TSH	thyroid stimulating hormone
